# 2,2′-[2,3,5,6-Tetra­methyl-*p*-phenyl­enebis­(methyl­eneoxy)]dibenzoic acid

**DOI:** 10.1107/S1600536810011220

**Published:** 2010-03-31

**Authors:** Tuoping Hu

**Affiliations:** a Department of Chemistry, North University of China, Taiyuan, Shanxi 030051, People’s Republic of China

## Abstract

The asymmetric unit of the title compound, C_26_H_26_O_6_, contains only a half-mol­ecule, the other half being generated by an inversion center. The two carboxy­phenoxy­methyl units occupy the 1,4-positions of the central aromatic ring. The central ring and the six linked C atoms are almost planar, with a maximum deviation of 0.0286 (17) Å, and the plane makes a dihedral angle of 75.50 (6)° with the benzene ring. In the crystal, strong O—H⋯O hydrogen bonds between the carboxyl groups of adjacent mol­ecules and C—H⋯π inter­actions link the mol­ecules into zigzag chains along (220) and (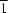
10); the two types of chain are arranged alternately, forming a three-dimensional framework.

## Related literature

For a structure with a similar central ring, see: Britton (2003 ▶). For structures with similar hydrogen-bonded carboxyl­ate groups, see: Bailey & Brown (1967 ▶); Glidewell *et al.* (2004 ▶).
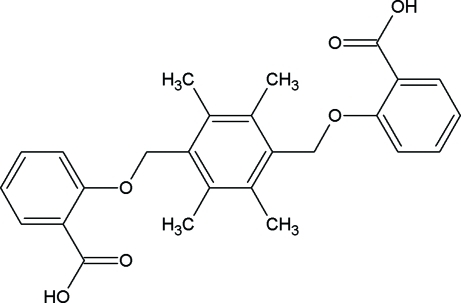


## Experimental

### 

#### Crystal data

C_26_H_26_O_6_
*M* *_r_* = 434.47
Monoclinic, 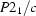

*a* = 9.2841 (16) Å
*b* = 8.6936 (15) Å
*c* = 14.075 (2) Å
β = 96.902 (3)°
*V* = 1127.8 (3) Å^3^
*Z* = 2
Mo *K*α radiation
μ = 0.09 mm^−1^
*T* = 293 K
0.16 × 0.12 × 0.06 mm

#### Data collection

Bruker SMART CCD area-detector diffractometer
Absorption correction: multi-scan (*SADABS*; Sheldrick, 1996 ▶) *T*_min_ = 0.985, *T*_max_ = 0.994
6437 measured reflections
2322 independent reflections
1269 reflections with *I* > 2σ(*I*)
*R*_int_ = 0.033

#### Refinement

*R*[*F* ^2^ > 2σ(*F* ^2^)] = 0.050
*wR*(*F* ^2^) = 0.157
*S* = 1.02
2322 reflections
159 parameters
H-atom parameters constrained
Δρ_max_ = 0.23 e Å^−3^
Δρ_min_ = −0.15 e Å^−3^

### 

Data collection: *SMART* (Bruker, 2007 ▶); cell refinement: *SAINT-Plus* (Bruker, 2007 ▶); data reduction: *SAINT-Plus*; program(s) used to solve structure: *SHELXS97* (Sheldrick, 2008 ▶); program(s) used to refine structure: *SHELXL97* (Sheldrick, 2008 ▶); molecular graphics: *SHELXTL* (Sheldrick, 2008 ▶); software used to prepare material for publication: *SHELXTL*.

## Supplementary Material

Crystal structure: contains datablocks global, I. DOI: 10.1107/S1600536810011220/bq2194sup1.cif

Structure factors: contains datablocks I. DOI: 10.1107/S1600536810011220/bq2194Isup2.hkl

Additional supplementary materials:  crystallographic information; 3D view; checkCIF report

## Figures and Tables

**Table 1:** Hydrogen-bond geometry (Å, °) *Cg*1 is the centroid of the central C9,C10,C12′,C9′C10′,C12 ring.

*D*—H⋯*A*	*D*—H	H⋯*A*	*D*⋯*A*	*D*—H⋯*A*
O2*A*—H2*A*⋯O1^i^	0.82	1.83	2.624 (9)	164
O2*B*—H2*B*⋯O1^i^	0.82	1.91	2.721 (16)	168
C5—H5⋯*Cg*1^ii^	0.93	2.80	3.356 (5)	120

Symmetry codes: (i) 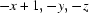; (ii) 
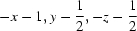
.

**Fig. 1.**
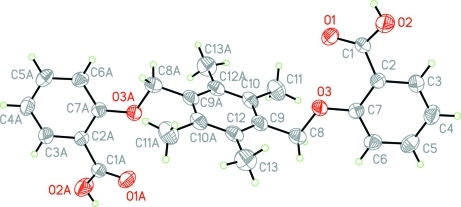
Molecular structure showing 50% probability displacement ellipsoids. The atoms marked with A are derived from the reference atoms by means of the (- x, 1 - y, - z) symmetry transformation..

**Fig. 2.**
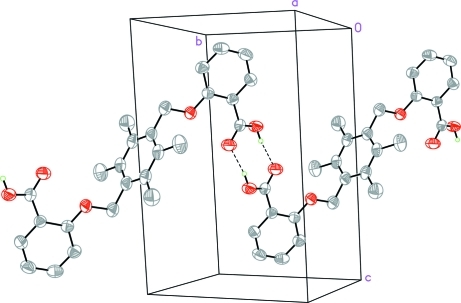
Zig-zag chain along (220) direction in the structure. Strong hydrogen bondings are shown as dash lines.

**Fig. 3.**
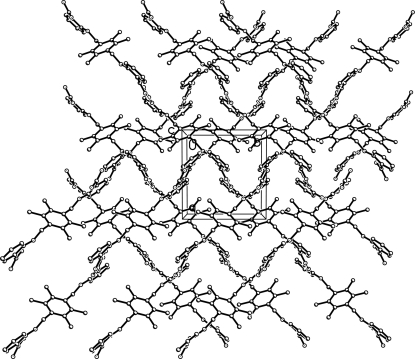
Packing diagram viewed down the c axis.

## supplementary crystallographic information

### Comment

The title compound (I), was designed as a ligand for preparing MOF materials. This paper reports its single crystal structure in the solid state. The asymmetric unit contains only half of the molecule, with the other half generated by an inversion center at (0,1/2,0). (Fig 1.). The two branches of benzenic-carboxylate acid groups of the title compound occupy the 1, 4 positions of the central aromatic ring to form a line. The central ring with its linked six C atoms, similar to the one observed in Britton (2003), is almost planar, with a maxima deviation of -0.0286 (17)° for C13 and makes a dihedral angle of 75.50 (6)° with the benzene ring. Strong C–H···π bond was observed in the structure, with a perpendicular distance of 2.700 (3)° to the C9 C10-C12-C9_i_C10_i_C12_i_ ring plane (i:-x, -y, -z). Meanwhile, the structure of the two carboxylate groups linked by the strong H-bond (Table 1) is comparable to that described in Bailey & Brown (1967) and Glidewell *et al.* (2004). Strong hydrogen bondings between the carboxylate groups of the adjacent molecules link the molecules of title compound into zig-zag chains along (220) and (-110) directions, respectively (Fig. 2), and these two chains were arranged alternatively to form a 3D framework (Fig. 3).

### Experimental

1,4-bis(bromomethyl)-2,3,5,6-tetramethylbenzene (3.2 g, 10.0 mmol), methyl salicylate (3.08 g, 22.0 mmol) and K_2_CO_3_ (3.04 g, 22 mmol) were put into 40 ml of acetone and the mixture was heated to reflux for 6 hours. The resulting mixture was filtrated while it was still hot. Lots of white precipitate was formed when the filtrate was cooled down to room temperature. The precipitate was filtrated and was put into 30 ml methanol. NaOH aqueous solution (20 ml, 2 mol/L) was added and the solution was stirred for 8 hours under refluxing. After cooling to room temperature, the pH value of the clear solution was adjusted to 2 by dilute hydrochloric acid. The clear solution was allowed to evaporate slowly under inert atmosphere. Prismatic crystals of the title compound were obtained after 2 days. The crystals were filtered, washed by cold EtOH and dried in air.

### Refinement

All H atoms were positioned geometrically and refined using a riding model with C—H = 0.930 Å and *U*_iso_(H) = 1.2 *U*_eq_(C) for aromatic hydrogens, and with C—H = 0.960 Å and *U*_iso_(H) = 1.5 *U*_eq_(C) for H atoms of the methyl groups, and with C—H = 0.970 Å and *U*_iso_(H) = 1.2 *U*_eq_(C) for H atoms of the methylene group, and with O—H = 0.820 Å and *U*_iso_(H) = 1.2 *U*_eq_(O) for the hydroxide H atom, respectively. O2 atom was treated as disordered in two positions of O2a and O2b with the occupation factors of 0.58, and 0.42, respectively.

### Crystal data

**Table:**

C_26_H_26_O_6_	*F*(000) = 460
*M**_r_* = 434.47	*D*_x_ = 1.279 Mg m^−^^3^
Monoclinic, *P*2_1_/*c*	Mo *K*α radiation, λ = 0.71073 Å
Hall symbol: -P 2ybc	Cell parameters from 1055 reflections
*a* = 9.2841 (16) Å	θ = 2.8–27.5°
*b* = 8.6936 (15) Å	µ = 0.09 mm^−^^1^
*c* = 14.075 (2) Å	*T* = 293 K
β = 96.902 (3)°	Prism, colorless
*V* = 1127.8 (3) Å^3^	0.16 × 0.12 × 0.06 mm
*Z* = 2	

### Data collection

**Table:**

Bruker SMART CCD area-detector diffractometer	2322 independent reflections
Radiation source: fine-focus sealed tube	1269 reflections with *I* > 2σ(*I*)
graphite	*R*_int_ = 0.033
phi and ω scans	θ_max_ = 27.5°, θ_min_ = 2.2°
Absorption correction: multi-scan (*SADABS*; Sheldrick, 1996)	*h* = −10→12
*T*_min_ = 0.985, *T*_max_ = 0.994	*k* = −11→11
6437 measured reflections	*l* = −16→18

### Refinement

**Table:**

Refinement on *F*^2^	Primary atom site location: structure-invariant direct methods
Least-squares matrix: full	Secondary atom site location: difference Fourier map
*R*[*F*^2^ > 2σ(*F*^2^)] = 0.050	Hydrogen site location: inferred from neighbouring sites
*wR*(*F*^2^) = 0.157	H-atom parameters constrained
*S* = 1.02	*w* = 1/[σ^2^(*F*_o_^2^) + (0.0659*P*)^2^ + 0.1641*P*] where *P* = (*F*_o_^2^ + 2*F*_c_^2^)/3
2322 reflections	(Δ/σ)_max_ < 0.001
159 parameters	Δρ_max_ = 0.23 e Å^−^^3^
0 restraints	Δρ_min_ = −0.15 e Å^−^^3^

### Special details

**Table:**

Geometry. All esds (except the esd in the dihedral angle between two l.s. planes) are estimated using the full covariance matrix. The cell esds are taken into account individually in the estimation of esds in distances, angles and torsion angles; correlations between esds in cell parameters are only used when they are defined by crystal symmetry. An approximate (isotropic) treatment of cell esds is used for estimating esds involving l.s. planes.
Refinement. Refinement of F^2^ against ALL reflections. The weighted R-factor wR and goodness of fit S are based on F^2^, conventional R-factors R are based on F, with F set to zero for negative F^2^. The threshold expression of F^2^ > 2sigma(F^2^) is used only for calculating R-factors(gt) etc. and is not relevant to the choice of reflections for refinement. R-factors based on F^2^ are statistically about twice as large as those based on F, and R- factors based on ALL data will be even larger.

### Fractional atomic coordinates and isotropic or equivalent isotropic displacement parameters (Å^2^)

**Table:**

	*x*	*y*	*z*	*U*_iso_*/*U*_eq_	Occ. (<1)
C1	0.4303 (2)	0.0417 (3)	0.12146 (17)	0.0593 (6)	
C2	0.3798 (2)	0.0589 (3)	0.21720 (15)	0.0552 (6)	
C3	0.4391 (3)	−0.0387 (3)	0.29015 (17)	0.0711 (7)	
H3	0.5065	−0.1123	0.2768	0.085*	
C4	0.4006 (3)	−0.0291 (3)	0.38111 (18)	0.0820 (8)	
H4	0.4405	−0.0963	0.4286	0.098*	
C5	0.3027 (3)	0.0805 (3)	0.40112 (17)	0.0775 (8)	
H5	0.2774	0.0885	0.4629	0.093*	
C6	0.2413 (3)	0.1787 (3)	0.33116 (17)	0.0666 (7)	
H6	0.1750	0.2526	0.3459	0.080*	
C7	0.2781 (2)	0.1680 (3)	0.23858 (15)	0.0558 (6)	
C8	0.1090 (3)	0.3686 (3)	0.18341 (18)	0.0764 (8)	
H8A	0.1458	0.4480	0.2283	0.092*	
H8B	0.0318	0.3139	0.2097	0.092*	
C9	0.0536 (3)	0.4386 (3)	0.08832 (18)	0.0666 (7)	
C10	−0.0399 (3)	0.3513 (3)	0.0242 (2)	0.0687 (7)	
C11	−0.0835 (3)	0.1899 (3)	0.0519 (2)	0.1008 (10)	
H11A	−0.1073	0.1293	−0.0048	0.151*	
H11B	−0.1664	0.1959	0.0865	0.151*	
H11C	−0.0043	0.1428	0.0916	0.151*	
C12	0.0955 (2)	0.5869 (3)	0.06413 (19)	0.0687 (7)	
C13	0.2028 (3)	0.6782 (4)	0.1319 (2)	0.1005 (10)	
H13A	0.2806	0.7135	0.0983	0.151*	
H13B	0.2412	0.6137	0.1843	0.151*	
H13C	0.1545	0.7649	0.1560	0.151*	
O1	0.3567 (2)	0.0876 (2)	0.04745 (12)	0.0919 (7)	
O2A	0.5605 (9)	−0.0059 (16)	0.1171 (6)	0.076 (3)	0.59 (4)
H2A	0.5699	−0.0296	0.0618	0.114*	0.59 (4)
O2B	0.524 (3)	−0.059 (4)	0.1191 (11)	0.130 (6)	0.41 (4)
H2B	0.5576	−0.0543	0.0678	0.196*	0.41 (4)
O3	0.22344 (16)	0.26410 (18)	0.16631 (11)	0.0674 (5)	

### Atomic displacement parameters (Å^2^)

**Table:**

	*U*^11^	*U*^22^	*U*^33^	*U*^12^	*U*^13^	*U*^23^
C1	0.0584 (15)	0.0585 (15)	0.0617 (15)	0.0095 (12)	0.0096 (12)	−0.0021 (11)
C2	0.0541 (13)	0.0586 (13)	0.0532 (13)	0.0005 (11)	0.0072 (10)	−0.0003 (10)
C3	0.0738 (16)	0.0709 (16)	0.0683 (16)	0.0109 (13)	0.0072 (13)	0.0078 (13)
C4	0.094 (2)	0.092 (2)	0.0604 (16)	0.0041 (17)	0.0079 (14)	0.0189 (14)
C5	0.0815 (18)	0.096 (2)	0.0577 (15)	−0.0132 (16)	0.0189 (14)	0.0057 (15)
C6	0.0649 (15)	0.0753 (17)	0.0631 (15)	−0.0003 (13)	0.0218 (12)	−0.0003 (13)
C7	0.0540 (13)	0.0596 (14)	0.0553 (13)	−0.0030 (11)	0.0122 (10)	0.0025 (11)
C8	0.0746 (16)	0.0790 (18)	0.0793 (17)	0.0215 (14)	0.0239 (14)	−0.0003 (14)
C9	0.0591 (14)	0.0666 (16)	0.0768 (16)	0.0177 (12)	0.0189 (13)	0.0001 (13)
C10	0.0618 (15)	0.0567 (15)	0.0899 (18)	0.0113 (12)	0.0192 (14)	0.0024 (14)
C11	0.100 (2)	0.072 (2)	0.130 (3)	−0.0029 (16)	0.0143 (19)	0.0104 (17)
C12	0.0569 (14)	0.0634 (16)	0.0872 (18)	0.0098 (12)	0.0143 (13)	−0.0102 (14)
C13	0.092 (2)	0.094 (2)	0.112 (2)	−0.0043 (18)	0.0007 (18)	−0.0085 (18)
O1	0.0932 (13)	0.1265 (17)	0.0561 (11)	0.0414 (12)	0.0097 (10)	0.0044 (10)
O2A	0.062 (4)	0.116 (6)	0.052 (3)	0.028 (3)	0.011 (3)	0.003 (3)
O2B	0.160 (11)	0.133 (12)	0.115 (8)	0.077 (9)	0.088 (7)	0.054 (6)
O3	0.0686 (10)	0.0736 (11)	0.0631 (10)	0.0220 (8)	0.0207 (8)	0.0080 (8)

### Geometric parameters (Å, °)

**Table:**

C1—O2B	1.237 (16)	C8—H8A	0.9700
C1—O1	1.241 (3)	C8—H8B	0.9700
C1—O2A	1.286 (9)	C9—C10	1.399 (3)
C1—C2	1.487 (3)	C9—C12	1.401 (3)
C2—C3	1.393 (3)	C10—C12^i^	1.395 (3)
C2—C7	1.397 (3)	C10—C11	1.524 (4)
C3—C4	1.372 (3)	C11—H11A	0.9600
C3—H3	0.9300	C11—H11B	0.9600
C4—C5	1.369 (4)	C11—H11C	0.9600
C4—H4	0.9300	C12—C10^i^	1.395 (3)
C5—C6	1.375 (3)	C12—C13	1.518 (4)
C5—H5	0.9300	C13—H13A	0.9600
C6—C7	1.389 (3)	C13—H13B	0.9600
C6—H6	0.9300	C13—H13C	0.9600
C7—O3	1.366 (3)	O2A—H2A	0.8200
C8—O3	1.440 (3)	O2B—H2B	0.8200
C8—C9	1.503 (3)		
			
O2B—C1—O1	121.6 (9)	C9—C8—H8B	110.4
O1—C1—O2A	119.3 (5)	H8A—C8—H8B	108.6
O2B—C1—C2	113.3 (7)	C10—C9—C12	120.8 (2)
O1—C1—C2	121.8 (2)	C10—C9—C8	118.3 (2)
O2A—C1—C2	118.4 (4)	C12—C9—C8	120.9 (2)
C3—C2—C7	118.2 (2)	C12^i^—C10—C9	120.0 (2)
C3—C2—C1	117.9 (2)	C12^i^—C10—C11	120.1 (2)
C7—C2—C1	124.0 (2)	C9—C10—C11	119.8 (2)
C4—C3—C2	121.8 (2)	C10—C11—H11A	109.5
C4—C3—H3	119.1	C10—C11—H11B	109.5
C2—C3—H3	119.1	H11A—C11—H11B	109.5
C5—C4—C3	119.2 (2)	C10—C11—H11C	109.5
C5—C4—H4	120.4	H11A—C11—H11C	109.5
C3—C4—H4	120.4	H11B—C11—H11C	109.5
C4—C5—C6	120.9 (2)	C10^i^—C12—C9	119.2 (2)
C4—C5—H5	119.6	C10^i^—C12—C13	120.2 (3)
C6—C5—H5	119.6	C9—C12—C13	120.6 (3)
C5—C6—C7	120.2 (2)	C12—C13—H13A	109.5
C5—C6—H6	119.9	C12—C13—H13B	109.5
C7—C6—H6	119.9	H13A—C13—H13B	109.5
O3—C7—C6	123.1 (2)	C12—C13—H13C	109.5
O3—C7—C2	117.07 (19)	H13A—C13—H13C	109.5
C6—C7—C2	119.8 (2)	H13B—C13—H13C	109.5
O3—C8—C9	106.66 (18)	C1—O2A—H2A	109.5
O3—C8—H8A	110.4	C1—O2B—H2B	109.5
C9—C8—H8A	110.4	C7—O3—C8	118.47 (17)
O3—C8—H8B	110.4		
			
O2B—C1—C2—C3	−2(2)	C3—C2—C7—C6	1.4 (3)
O1—C1—C2—C3	158.1 (2)	C1—C2—C7—C6	−177.8 (2)
O2A—C1—C2—C3	−30.0 (8)	O3—C8—C9—C10	78.2 (3)
O2B—C1—C2—C7	178 (2)	O3—C8—C9—C12	−101.3 (2)
O1—C1—C2—C7	−22.7 (4)	C12—C9—C10—C12^i^	−1.6 (4)
O2A—C1—C2—C7	149.2 (8)	C8—C9—C10—C12^i^	179.0 (2)
C7—C2—C3—C4	−0.4 (4)	C12—C9—C10—C11	179.7 (2)
C1—C2—C3—C4	178.9 (2)	C8—C9—C10—C11	0.3 (3)
C2—C3—C4—C5	−0.8 (4)	C10—C9—C12—C10^i^	1.5 (4)
C3—C4—C5—C6	1.0 (4)	C8—C9—C12—C10^i^	−179.0 (2)
C4—C5—C6—C7	0.0 (4)	C10—C9—C12—C13	−177.0 (2)
C5—C6—C7—O3	−178.6 (2)	C8—C9—C12—C13	2.5 (3)
C5—C6—C7—C2	−1.3 (3)	C6—C7—O3—C8	−8.1 (3)
C3—C2—C7—O3	178.9 (2)	C2—C7—O3—C8	174.5 (2)
C1—C2—C7—O3	−0.3 (3)	C9—C8—O3—C7	−170.4 (2)

Symmetry codes: (i) −*x*, −*y*+1, −*z*.

### Hydrogen-bond geometry (Å, °)

**Table:**

Cg1 is the centroid of the central C9,C10,C12',C9'C10',C12 ring.

**Table:**

*D*—H···*A*	*D*—H	H···*A*	*D*···*A*	*D*—H···*A*
O2A—H2A···O1^ii^	0.82	1.83	2.624 (9)	164
O2B—H2B···O1^ii^	0.82	1.91	2.721 (16)	168
C5—H5···Cg1^iii^	0.93	2.80	3.356 (5)	120

Symmetry codes: (ii) −*x*+1, −*y*, −*z*; (iii) −*x*−1, *y*−1/2, −*z*−1/2.
